# Surgical Treatment Intensity at the End of Life in Patients With Cancer: A Systematic Review

**DOI:** 10.1097/AS9.0000000000000514

**Published:** 2024-11-12

**Authors:** Samuel Lawday, Benjamin E. Zucker, Shona Gardner, James Robb, Lorna Leandro, William Hollingworth, Jane Blazeby, Angus G.K. McNair, Charlotte Chamberlain

**Affiliations:** From the * NIHR Bristol Biomedical Research Centre, University Hospitals Bristol and Weston NHS Foundation Trust and University of Bristol, Bristol, UK; † Department of General Surgery, North Bristol NHS Trust, Bristol, UK; ‡ Population Health Sciences, Bristol Medical School, University of Bristol, Canynge Hall, Bristol, UK; § Division of Surgery, University Hospitals Bristol NHS Foundation Trust, Bristol, UK; ‖ Population Health Science, UK Palliative and End of Life Care Research Group, Bristol Medical School, Bristol, UK; ¶ Department of Palliative Care, University Hospitals Bristol and Weston NHS Foundation Trust, Bristol, UK

**Keywords:** end of life, review, surgery, treatment Intensity

## Abstract

**Objective:**

To synthesize evidence of surgical treatment intensity, defined as a measure of the quantity of invasive procedures, received by patients in patients with cancer within a defined time period around the ‘end of life’ (EoL).

**Background:**

Concern regarding overly ‘aggressive’ care or high health care utilization at the EoL, particularly in cancer, is growing. The contribution surgery makes to the quality and cost of EoL care in cancer has not yet been quantified.

**Methods:**

This PROSPERO registered systematic review used PRIMSA guidelines to search electronic databases for observational studies detailing surgical intensity at the EoL in adult cancer patients. Intensity was compared by disease, individual characteristics, geographical region, and palliative care involvement. A risk of bias tool assessed quality and a narrative synthesis of findings was completed.

**Results:**

In total, 39 papers were identified in this search. Up to 79% of patients underwent invasive procedures in the last month of life. Heterogeneity in patient groups, inclusion criteria, and EoL time periods lead to huge variation in results, with treatment intention often not identified. Patient, geographical, and pathological factors, alongside involvement of palliative/hospice care, were all identified as contributors to treatment intensity variation.

**Conclusions:**

A significant proportion of patients with cancer undergo invasive and costly invasive procedures at the EoL. There is significant reporting heterogeneity, with variation in patient inclusion criteria and EoL timeframes, demonstrating uncertainty within the literature. Identification of the context where surgical treatment intensity at the EoL is potentially inappropriate is not currently possible.

## INTRODUCTION

Up to one-third of patients undergo an operation in their last year of life.^1^ Many of these operations proceed with the intent of prolonging life or relieving distressing symptoms. However, invasive procedures at the end of life (EoL) may reflect inappropriate care.^2^ It is estimated that 33% to 38% of patients near the EoL receive nonbeneficial (little or no chance of benefit or for which the risks outweigh the benefits^3^) treatments, though the contribution of surgery to this figure is unclear.^2,4^ Healthcare costs in the last year of life may be up to 5 times greater than in other years, with 13% of US healthcare expenditure spent on individuals in their last year of life, though again the contribution of surgery to this figure is unclear.^5,6^

Different factors affect EoL treatment intensity. Patients who are younger, have a higher household income and have specific underlying diagnoses have increased treatment intensity in the last 90 days of life.^7,8^ Variation in treatment intensity by geographical area has also been identified.^9,10^ A low number of nursing home beds and treatment in a tertiary-care setting are risk factors for increased treatment intensity at the EoL.^11^ Conversely, palliative care referrals have been linked to reduced treatment intensity.^12^

High-quality research in surgical care at the EoL is lacking, reflected by its inclusion in National Health Service England’s research needs assessment.^13^ Studies have demonstrated that systemic anticancer and radiotherapeutic interventions may be ineffective or harmful, costly, and not in line with patients’ wishes towards the EoL.^14^ Despite evidence of the growing intensity of surgical treatment in the last year of life, there has been little focus on what contribution surgery makes to the quality and cost of EoL care.^15^

Evaluating surgical treatment intensity at the EoL is challenging. There is variation in the definition of surgical intensity, the EoL timeframe used, and understanding of procedural intent, which makes summation of this data challenging. Understanding the clinical or socioeconomic context where surgical treatment intensity at the EoL is potentially inappropriate is critical to addressing future policy and practice recommendations. This systematic search and narrative synthesis therefore aims to report on surgical treatment intensity in the EoL period according to patient demographics, disease type, EoL time frame, geographical region, and involvement of palliative and hospice care to understand current practice.

## METHODS

This systematic review was registered with Prospero [CRD42020173306] and reported according to Preferred Reporting Items for Systematic reviews and Meta-Analyses guidelines.^16^

### Search Strategy

A systematic electronic database search of OVID SP versions of MEDLINE and Embase, the Cochrane Database of Systematic Reviews, and the Cochrane Central Register of Controlled Trials was undertaken in April 2023 to identify articles addressing the intensity of surgical care at the EoL published since 2014. Papers published between 1990 and 2014 were identified through a previous systematic review and were brought forward for screening.^4^ Supplementary snowball searches from reference lists of included studies were completed and a modified search was repeated after identification of articles not included in the original search. Endnote software was used to organize and store included studies.

### Eligibility Criteria

Included articles reported surgical intensity in a self-defined EoL period in patients over the age of 18 with solid organ tumors. Comparative studies, cohort, or case series study designs were included. Randomized controlled trials, case reports, letters, reviews, or comments were excluded. Exclusion criteria were studies including patients solely under 18 years old, or solely hematological or skin cancer and non-English language publications (Table 1).

**TABLE 1.** Inclusion and Exclusion Criteria

Inclusion Criteria	Exclusion Criteria
Cohort, case–control, ecological, and clinical practice audit studies	Studies of populations less than 18 years of age
Measured surgical intensity defined as a measure of the quantity of invasive procedures received by patients (such as frequency, rate, or cost)	Only nonsolid organ malignancies (hematological and nonmelanoma skin malignancy)
A defined EoL time period	

### Screening and Data Extraction

Four authors screened titles and abstracts according to predefined eligibility criteria (S.L., B.E.Z., S.G., and L.L.). Five authors completed full-text screening of shortlisted studies independently (S.L., B.E.Z., S.G., L.L., and J.R.), with disagreements resolved through discussion with senior team members (A.G.K.M. and C.C.). Data were extracted into a standardized, prepiloted data extraction form by 4 authors with a 10% sample dual extracted (Supplementary Appendix 1, http://links.lww.com/AOSO/A421) (S.L., B.E.Z., S.G., and J.R.).

Predefined outcomes of interest were entered into an Excel spreadsheet (Microsoft, US). These included article details (eg, author, name of article, country, and study type), patient demographics (eg, number, gender, age, underlying pathology, demographics, and deprivation indices), and surgical intensity outcomes. This included the invasive procedures undertaken, the measurement of procedural intensity, and variation in procedural intensity with any comparators from the article (eg, cancer type and palliative care involvement). Analysis strategy, cost analysis, and effect measure used were also extracted from each included publication.

### Narrative Synthesis

Meta-analysis was not feasible due to the heterogeneity of the time frames, cancer populations and outcome measures. Narrative synthesis was performed using comparative tables.^17^ Reported specific invasive procedures were categorized according to British United Provident Association (BUPA) classifications.

### Risk of Bias

A risk of bias analysis was completed for each included study.^18^ This described the quality of included studies. No studies were excluded based on methodological quality. A single author did this with 10% dual assessed (S.L., S.G., B.E.Z., and J.R.).

## RESULTS

### Preferred Reporting Items for Systematic reviews and Meta-Analyses Diagram

**FIGURE 1.**
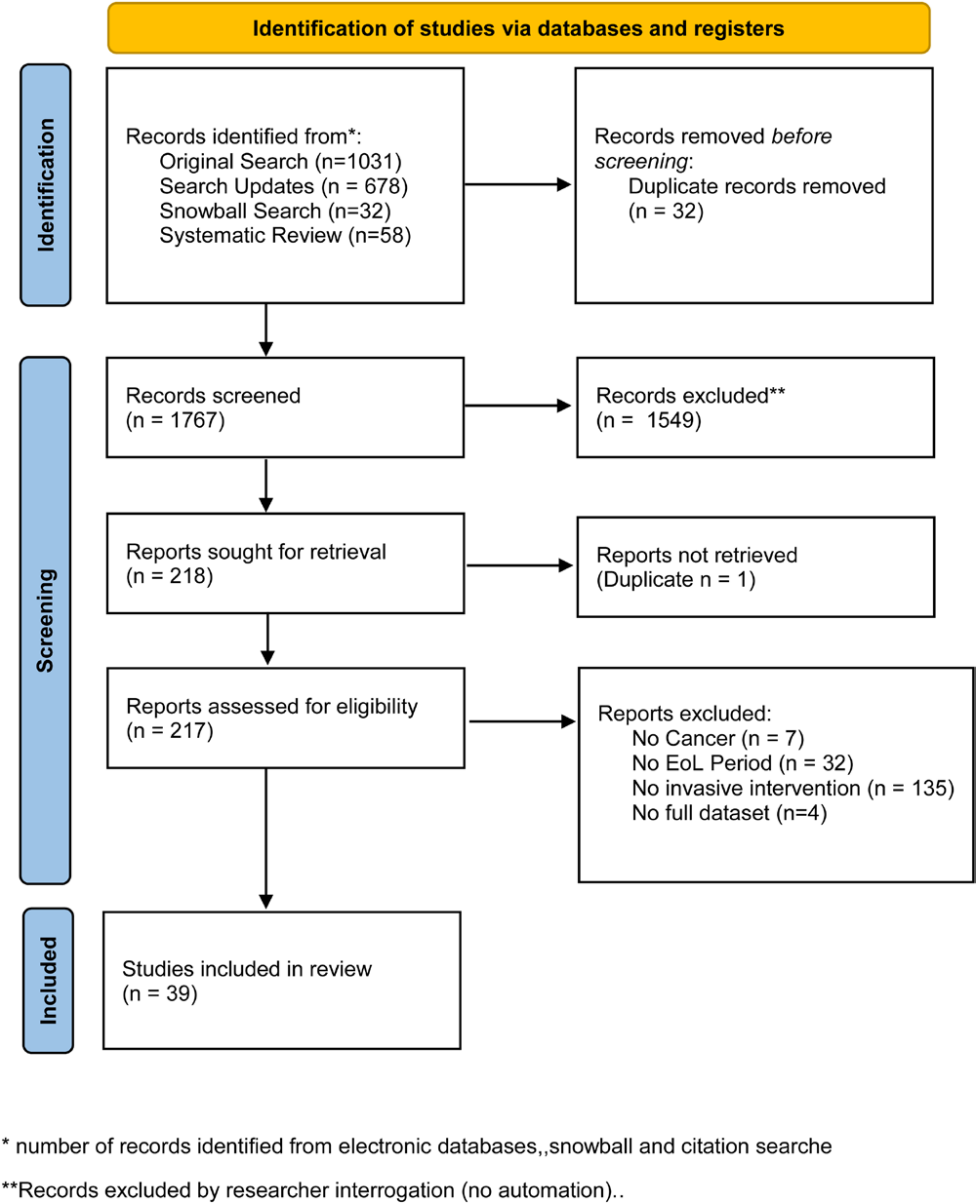
Preferred Reporting Items for Systematic Reviews and Meta-Analyses flow diagram. *number of records identified from electronic databases, snowball, and citation searches. **Records excluded by researcher interrogation (no automation). Adapted with permission from^16^.

The search yielded 1767 abstracts, of which 218 were eligible for full-text screening (Figure 1). However, 39 papers were included in this review^1,15,19–55^ (Tables 2 and 3).

**TABLE 2.** Included Papers

Author	Year	Country	Journal	Study Type	Cancer Types	Patients	Decedents
Axelsson	1997	Sweden	European Journal of Surgical Oncology	Cohort	Mixed cancers	208	208
Barnato	2004	USA	Health Services Research	Case–Controlled	Mixed cancers (and noncancers)	2,913,708	541,697
Braga	2007	Portugal	Psycho-Oncology	Cohort	Mixed cancers	319	319
Kwok	2011	USA	Lancet	Cohort	Mixed cancers (and noncancers)	1,802,029	1,802,029
Barnet	2013	USA	Anesthesiology	Case–Controlled	Mixed cancers (and noncancers)	747	37
Lopez	2013	USA	Gynecologic Oncology	Cohort	Gynecological	220	220
Kwok	2015	USA	Journal of Surgical Research	Cohort	Mixed cancers	100594	Unspecified
Alturki	2014	Canada	Journal of Neuro-Oncology	Cohort	Neurological	1623	1623
Obermeyer	2014	USA	JAMA	Case–Controlled	Mixed cancers (and noncancers)	36330	36330
Collins	2014	Australia	Journal of Neuro-Oncology	Cohort	Neurological	1160	1160
Krell	2015	USA	Cancer	Cohort	Colorectal cancer	84161	Unspecified
Shiovitz	2015	USA	Cancer epidemiology, biomarkers and prevention	Cohort	Mixed cancers	76,259	64,244
Wu	2015	USA	Journal of Oncology Practice	Cohort	Mixed cancers	116	116
Barnato	2015	USA	Journal of Pain and Symptom Management	Case–Controlled	Mixed cancers (and noncancers)	173,044	120,372
Du	2015	USA	Lung Cancer	Cohort	Lung cancer	37,393	37,393
Liu	2016	Taiwan	Annals of Surgery	Cohort	Mixed cancers	339,546	339,546
Ong	2017	Australia	Asia-pacific Journal of Clinical Oncology	Cohort	Mixed cancers	27,926	27,926
Daly	2016	USA	Journal of Oncology Practice	Cohort	Mixed cancers	72	72
Triplett	2017	USA	Journal of Oncology Practice	Cohort	Mixed cancers	6580	6580

**TABLE 3.** Included Papers

Author	Year	Country	Journal	Study Type	Cancer Types	Patients	Decedents
Tukey	2018	USA	Journal of Palliative Medicine	Cohort	Lung cancer	19,930	19,930
Schwartz	2018	USA	Cancer	Cohort	Breast cancer (and noncancer)	1244	833
Jang	2018	South Korea	BMC Palliative Care	Cohort	Gynecological Cancer	193	193
Sompratthana	2018	Thailand	International Journal of Gynaecological Cancer	Cohort	Gynecological Cancers	159	159
Urban	2018	USA	Gynecologic Oncology	Cohort	Gynecological Cancers	5509	5509
Kuo	2019	Taiwan	Journal of pain and symptom management	Case–Controlled	Lung cancer	11,937	11,937
De Man	2019	Netherlands	Cancer Control	Cohort	Mixed cancers	40,444	40,444
Niteki	2019	USA	International Journal of Gynaecological Cancer	Cohort	Gynecological Cancers	391	391
Wächter	2020	Germany	European Archives of Oto-Rhino-Laryngology	Cohort	Thyroid cancer	42	40
Fond	2020	France	Journal of Affective Disorders	Case–control	Mixed cancers (and noncancers)	226,547	226,547
Fond	2020	France	American Psychosomatic Society	Case–Controlled	Mixed cancers (and noncancers)	224,492	224,492
Martins-Brance	2020	Portugal	European Society for Medical Oncology	Cohort	Mixed cancers	92,155	92,155
Viprey	2021	France	European Archives of Psychiatry and Clinical Neuroscience	Case–Controlled	Lung cancer	67,102	67,102
Fond	2021	France	Nature	Cohort	Breast cancer	38,612	38,612
Schmitz	2021	Netherlands	Cancers	Cohort	Breast cancer	203	203
Ullgren	2021	Sweden	PLOS ONE	Cohort	Lung or pancreatic Cancer	1726	1726
Wang	2022	Taiwan	International Journal of Environmental Research and Public Health	Cohort	Mixed cancers	8814	8814
Chiaruttini	2022	Italy	BMJ	Cohort	Mixed cancers	26,539	26,539
Broekman	2022	Netherlands	Gynecologic Oncology	Cohort	Gynecological Cancers	2172	2172
Vestergaard	2023	Denmark	Gynecologic Oncology	Cohort	Gynecological Cancers	4502	4502

### Study Characteristics

Of the 39 included papers, 20 studied participants with a range of cancer types. The median number of study participants was 8814 [42 (Wachter, Table 3) and 2,913,708 (Barnato, in Table 2)]. Eight of the studies included patients without cancer as a comparison group. Most studies were published after 2010 (37/39) and came from the United States (US) (17/39). There were no studies published from low-income countries. Twelve of the included articles were multicentre studies and the predominant study design was a cohort study (n = 31). Funding was described in 27 articles (Supplementary Table 1, http://links.lww.com/AOSO/A418) (Tables 4 and 5).

**TABLE 4.** Study Characteristics

	All studies (n = 39)
Publication year	
1997–2000	1
2001–2010	2
2011–2020	29
2021	7
Cancers	
Mixed cancer	21
Colorectal	1
Breast	3
Lung	4
Neuro	2
Gynaecological	7
Thyroid	1
Continent	
North America	18
Europe	14
Asia	5
Australasia	2
Centers	
National	14
Multicentre	12
Single center	13
Study design	
Cohort	31
Case–control	8

**TABLE 5.** Study Characteristics regarding EoL periods

Number of EoL Time Periods Measured	
1	22
2	8
3+	9
EoL time periods	
Fixed	
<1 month	13
<3 months	16
<6 months	14
<12 months	5
Clinical	
Admission to hospice	1
Diagnosis	5
Palliative care consultation	1
Last admission	2
Proportion of decedents	
100%	31
<100%	6
Unspecified	2

While most studies measured a single a priori-determined EoL time period (n = 22), 9 studies measured 3 or more EoL time periods. Eol time periods were defined heterogeneously. Some articles used a clinical event (eg, admission to hospice, first diagnosis, palliative care consultation, or admission with a life-limiting diagnosis), while others utilized a defined time period (ranging from days to under 1-year). While all 39 studies measured invasive procedural intensity by measuring the proportion of patients undergoing a procedure, a number of studies reported other measures as well; rates of procedure (n = 3), frequencies of procedure (n = 4), odds or risk ratios (n = 11) and other composite measurements, such as overall healthcare utilization or the EOL intensity index (n = 3).^56^

Seven studies considered cost associated with invasive procedural intensity of treatment at the EoL. They demonstrated higher costs associated with increased intervention in cancer patients during the EoL period, with higher costs close to the date of death.^1,26,27,40,44^ Only 1 study demonstrated the clear increased costs over time (from $10,567 to $16,320 between 1985 and 1999).^15^ Schwartz et al^30^ found that the majority of health care costs in triple-negative breast cancer occurred in the initial months of diagnosis and the last months before death.

### A Description of Invasive Procedures at the EoL in Included Studies

Two hundred twenty-nine procedure types were identified from 23 of the included studies and classified according to the BUPA system (Table 6). Seventy-four procedures were not reported in sufficient detail to be grouped according to the BUPA classification.

**TABLE 6.** BUPA Codes of Included Procedures

BUPA Code	n
Complex major	27
Major	50
Intermediate	46
Minor	36
Unclassified	74

There was significant heterogeneity in the specificity of intervention reporting. Liu et al^27^ used specific operative codes but also ‘diagnostic surgery’ and Tukey et al^33^ used ‘Other thoracic surgeries’, ‘Other major surgeries’, ‘Minimally invasive procedures’, and ‘Life-sustaining procedures’. Some articles simply referred to ‘surgery’ within an EoL period. The degree of invasiveness also varied significantly. Some articles included procedures that ranged from simple percutaneous procedures to major complex surgery. Frequently reported procedures in the EoL time periods included biopsies and gastrostomy insertion.

### Proportion of Patients Undergoing Invasive Procedure by Author Defined EoL Period

Table 7 summarizes the proportion of patients undergoing invasive procedures in articles that included over 1000 patients. Pooled data demonstrates that 17.3% of patients undergo an invasive procedure in the last month of life, though the variability between inclusion criteria of different studies means this is not reliable, and further analysis was therefore not completed. Over 10% of patients underwent invasive procedures in the last month of life in the 14 studies that reported on this.^1,19,26,27,33,35,37,44,46–49,53,55^ Two studies report that the intent of these procedures was most frequently ‘palliative’, although this was not defined but may reflect a mixture of minor and major procedures.^27,44^

**TABLE 7.** Proportion of Patients Undergoing Invasive Procedure by End of Life Period

Study	Cancer Type	Total Decedents	Time Periods ≤1 Month	Time Periods 1–3 Month	Time Periods 3–6 Month	Time Periods 6–12 Months
Barnato et al (2004)^15^	Mixed	541,697	-			141,239 (26.1%)
Kwok et al (2011)^1^	Mixed	1,802,029	329,771 (18.3%)	452,309 (25.1%)		575,596 (31.9%)
Alturki et al (2014)^19^	Neurosurgical	1623			252 (15.6%)	
Kwok et al (2015)^*^^26^	Mixed	100,594	25,551 (25.4%)			
Obermeyer et al (2014)^28^	Mixed	36,330				
Collins et al (2014)^38^	Neurosurgical	1160		61–121 (28–46%)		
Shiovitz et al (2015)^31^	Mixed	76,259				
Barnato et al (2015)^21^	Mixed	120,372				
Krell et al (2015)^24^	Colorectal	84,161				
Du et al (2015)^40^	Lung	37,393			1010 (2.7%)	
Liu et al (2016)^27^	Mixed	339,546	38,857 (11.4%)			
Ong et al (2017)^29^	Mixed	27,926	267 (1%)			
Triplett et al (2017)^32^	Mixed	6580	-			
Tukey et al (2018)^33^	Lung	19,930	3000 (15.1%)			
Schwartz et al (2018)^30^	Breast	1244				
Urban, et al (2018) ^44^	Gynecological	5509	573^†^ (10.4%)	1063^†^ (19.3%)		
Kuo et al (2019)^25^	Lung	3377			13 (0.38%)	
De Man et al (2019)^36^	Mixed	CRC = 14,911	343–641 (2.3%–4.3%)	1491–2088 (10%–14%)	2982–4026 (20%–27%)	
		Lung = 25,533	511–562 (2%–2.2%)	1787–1915 (7%–7.5%)	3575–3804 (14%–14.9%)	
Viprey, et al (2021)^44^	Lung	67,102	13346 (19.89%)			
Fond et al (2020)^37^ (JAD)	Mixed	20,320	3646 (17.9%)			
Fond, et al (2020 (APS)^46^	Mixed	10,050	3170 (31.5%)^‡^			
Martins-Branco, et al (2020)^47^	Mixed	92,155	25803^†^ (28%), 95% CI 28 to 28			
Fond et al (2021)^49^	Breast	38,612	4589 (11.88%)			
Ullgren, et al (2021)^51^	Mixed	1726	17 (2%)–14(1%)			
Wang et al (2022)^52^	Mixed	8814	786 (9.01%)			
Chiaruttini, et al (2022)^53^	Mixed	26,539	Minor—12 132 (45.7%), Major—1095 (4.1%)			
Broekman et al (2022)^54^	Gynaecological	2172	211^†^ 9.7% (8.2–11.1)			
Vestergaad et al (2023)^55^	Gynaecological	4502	49 (1.8), <5 (<0.2), <5 (<0.2)^*^			
Total	Mixed	3,518,166	17.33%	24.72%	10.99%	30.59%

For studies with >1000 patients.

* For different gynecological cancers

† Calculated from paper.

‡ 21% and 79% in unmatched groups.

CRC indicates colorectal cancer; LC, lung cancer; NB, if range present, higher number used and major>minor procedures selected.

Eight studies that reported on invasive procedures in the last 6 months to 1 year indicated that between 2.7% and 62% of patients underwent invasive procedures in this time frame, highlighting the huge variation reported within the literature. This, however, was 26.1% to 31.9% in the larger studies.^19,20,25,36,40,41,43,50^

Ten studies reported on the proportion of patients receiving invasive procedures in clinically defined time frames (Supplementary Table 2, http://links.lww.com/AOSO/A419).^21,24,26,28,30–32,34,38,43^ These included time from diagnosis, time from palliative/hospice care consultation, or time from admission with a life-limiting illness. Schwartz et al^30^ reported that 36.5% of those diagnosed with stage IV breast cancer underwent an invasive procedure between diagnosis and death, while Krell et al,^24^ found that 57.7% of patients diagnosed with metastatic colorectal cancer subsequently underwent primary site resection.^30^ Sompratthana et al^43^ identified 8.8% of patients with gynecological malignancy had an invasive procedure on their last admission before death.

### Intensity of Invasive Procedure by Patient Demographics

Few studies (7/39) compared treatment intensity at the EoL according to patient demographics. Age was identified as a factor, with treatment intensity lower among older patients.^1,27,33,47,54^ Ethnicity was reported to be associated with procedural treatment intensity, but the association was not consistent. Tukey et al^33^ showed that those with “nonwhite race” were more likely to undergo an invasive procedure in the last month of life [odds ratio = 1.244, confidence interval (CI) = 1.15–1.345] and Shiovitz et al^31^ also identified treatment intensity variation due to ethnicity with American Indian/Alaskan natives receiving less treatment than “non-Hispanic whites” [0.80 (0.74–0.86)].^31,33^ Du et al,^40^ after adjusting for numerous patient and disease characteristics, demonstrated black and minority ethnic populations received less surgery in the last 6 months of life (odds ratio = 0.77, CI = 0.57–1.04), when compared with the Caucasian population.^40^ Liu et al^27^ also identified that married, male patients had greater healthcare utilization at the EoL, but this was not assessed in other included studies^27^ (Tables 7 and 8).

**TABLE 8.** Variables Measured and Comparators for Surgical Intensity

	All studies (n = 39)
Demographics measured	
Age	39
Sex	31
Ethnicity	16
Patient comorbidity	24
Deprivation	11
Income	11
Geographical region	21
Primary cancer site	26
Procedures measured*	
All/unspecified	27
Defined group of procedures	13
Specific named procedures	13
Measure of surgical treatment intensity	
Rates of procedures	3
Frequency	4
Proportion of patients receiving procedures	39
Odds ratio/risk ratios	11
Costs	7
Other	3
Surgical treatment intensity comparisons	
Patient characteristics	7
Disease characteristics	10
Type of procedure	12
Geographic variation	10
Other†	21

* Defined groups of procedures were publication-defined groups (such as bowel resection or neurosurgical procedure), whereas specific named procedures were OPCS specific.

†‘ Other’ comprised a heterogeneous group of classifications by which surgical intensity was compared. This included the year of diagnosis or year of death, co-morbid status, exposure to palliative care or hospice, involvement in a trial, place of death, and stage of cancer.

### Intensity of Invasive Procedure by Cancer Type

Comparison of intervention intensity at the EoL by the cancer type was only completed in 7 articles. De Man et al,^36^ who demonstrated a greater proportion of those with colorectal cancer underwent invasive procedures than those with lung cancer.^36^ Liu et al^27^ studied >10 primary cancers and identified hepatic-pancreatic or lung cancers as those least likely to have intervention in the EoL period.^27^ This was supported by Martins-Branco 2020 who identified lung and colorectal cancer as most likely to have high treatment intensity at the EoL^47^ (Tables 7 and 8).

Other smaller studies found that those with gastro-intestinal malignancy were most likely to receive surgery within the last 6 months of life and those with breast cancer were the least likely, out of the 4 different cancer gr et al^27^oups considered.^20,39^ Daly et al^39^ measured only specific interventions, however, and so this may explain the difference in outcome compared to Martins-Branco.

### Intensity of Invasive Procedure and Geographical Variation

A measure of patient geography was included in 21 studies, however, treatment intensity according to geographical location was only explicitly discussed in 10 studies. The way geographical demographics were compared varied.

Geographical boundaries were used in 2 studies that considered the relationship between the intensity of invasive procedures at the EoL, with no variation identified.^33,40^

Geographical variation due to hospital proximity and healthcare capacity was used in different studies. Health care utilization at the EoL was greater in geographical regions with high healthcare capacity^1^ and in metropolitan areas.^40^ Proximity to hospital was also associated with increased hospital admissions.^20^

Hospital type was also identified by different articles as a factor in treatment intensity variation. Rates of invasive procedures were greater at the EoL for patients who received care in a teaching hospital or a hospital with more acute care hospital beds.^27^ Larger, urban, teaching hospitals also had higher EOL treatment intensity.^21^ There was also increased intervention for metastatic disease in higher volume centers.^24^

### Intensity of Invasive Procedures and Engagement with Palliative and Hospice Care

The relationship between hospice and palliative care and intensity at the EoL was discussed in 12 studies.^25,28,32,33,40,41,44,46,47,52,53,55^ Eleven of these identified lower associated healthcare costs and lower rates of invasive procedures associated with palliative care involvement. Obermeyer et al^28^ however, found patients without hospice involvement had higher healthcare costs by $8697 (95% CI = $7560–$9835) and were more likely to undergo invasive procedures.^28^

Du et al^40^ showed lower healthcare costs in the last 6 months of life for those who received hospice care, supporting these results.^40^ Triplett et al^32^ showed that after palliative care exposure, patients with metastatic cancer had reduced rates of all invasive procedures. Furthermore, patients exposed to palliative care were less likely to have more than 1 hospital admission, an intensive care admission, or die in acute care hospital. These results were further substantiated by those of Tukey et al^33^ who showed that among veterans with stage IV nonsmall cell lung cancer hospice utilization within the last month of life was strongly and negatively associated with receipt of invasive procedures. Vestergaard showed a reduction in surgery within 14 days of death in gynecological patients and Chairuttini demonstrated a reduction in both major and minor therapeutic procedures in all cancer patients in the month before death with involvement of palliative care.^53,55^

### Methodological Quality

High or moderate risk of bias was identified in 23 studies (high = 4, moderate = 19) and a low risk of bias in 16 studies. Bias was most commonly introduced due to the study sample being unrepresentative of the national population. For example, results from studies limited to the Veteran’s affairs database, or to specific geographical locations, were not generalizable. Data was often retrospective and used invalid or unreliable methods such as using hospital codes (Supplementary Table 3, http://links.lww.com/AOSO/A420).

## DISCUSSION

This systematic review demonstrates significant heterogeneity in the reporting practices within the published literature regarding surgical care at the EoL. Different patient groups, inclusion criteria and EoL time periods were used in the included articles, leading to huge variation in the results identified. Between 2.7% and 62% of patients underwent invasive procedures in the last 6 months to 1 year of life.^1,15,19,20,25,36^

Various factors were linked to variability in EoL surgical care. Certain cancers, namely lung and colorectal, were associated with higher rates of intervention.^27,47^ Patients who were younger were most likely to receive an invasive procedure, and patients’ ethnicity also had an impact on procedure intensity at the EoL. Geographical variation in practice was also identified, with procedural intensity at the EoL found to be higher in more populous areas,^40^ areas with high healthcare capacity^1^ and in larger or teaching hospitals.^27^ An inverse association between hospice or palliative care involvement and the frequency of invasive procedures and subsequent costs at the EoL was demonstrated.^25,28,32,33,40^ Included studies demonstrate the high cost associated with surgical intensity at the EoL.^15,25–27,40^

The EoL phase used varied between included articles, with articles using clinical time-points (eg, diagnosis) or a variety of fixed timeframes (eg, the last month of life). In addition to this, methods to systematically define treatment intent in routine data sources are deficient, which makes identifying procedural intent challenging. There was also variation in outcome measures used and patient inclusion criteria. Therefore, quantitative analysis was not possible and comparison between articles was challenging. There will inevitably be a cohort of included patients who had undergone treatment in their EoL period with curative intent; however, separating these out was not possible. The Risk of Bias Assessment Tool identified a number of low-quality studies and reflects the paucity of high-quality published data available to support frontline clinicians with their decision-making.

Reporting on the intensity of invasive procedures at the EoL is less prevalent than the intensity of chemotherapeutic regimens or intensive care admissions at the EoL, though the results show a similar trend.^4^ Studies have shown that between 7.1% and 16% of patients who were hospitalized with a diagnosis of metastatic cancer received chemotherapy in the last 14 days of life. The use of integrated and early palliative care to reduce the intensity of inappropriate care at the EoL has been suggested in other nonsurgical data, which is consistent with the results of this review.^9,57^ A 2014 study in noncancer patients identified reduced uptake of surgery and reduced patient mortality with the involvement of palliative care.^58^ It is possible that palliative care exposure aids in the formulation of clear treatment goals and accurate prognostication and therefore reduces the uptake of nonbeneficial surgery.^59^ However, it is also important to recognize the potential selection bias associated with palliative care referral in nonrandomized data. Those patients referred to palliative care may represent those less likely to be well enough for invasive procedures or reflect a difference in treatment intent with those who had undergone procedures being treated with curative intent.

The strength of this systematic review relates to its methodologically rigor, as it followed Cochrane systematic review guidance with prospective registration, dual screening of full-text articles, and assessment of the risk of bias. The study group included a medical librarian to improve the search strategy and members from surgery, palliative care, and health economics to improve the narrative synthesis. The main weakness of this systematic review is the article heterogeneity meaning that drawing clinical conclusions from this article is not possible. From the results in this review, it is not possible to conclude whether there is surgical overtreatment at the EoL. Articles did not identify procedural intent and with the assorted EoL periods and inclusion criteria used, drawing true clinical outcomes is not possible. It does, however, highlight the need for well-structured formal research in this area with the creation of reporting guidelines to increased standardization.

## CONCLUSIONS

This review summarizes and appraises the evidence of invasive procedures at the EoL. There is significant reporting heterogeneity, with variation in patient inclusion criteria and EoL timeframes, demonstrating uncertainty within the literature. The results in the review do, however, reflect significant practice variation, again likely due to poor evidence in this area. Conclusions as to whether there is surgical overtreatment at the EoL cannot be made. However, it is clear surgical care intensity at the EoL is high and financially costly. There is significant variation due to cancer, patient, and geographical factors. While palliative care involvement may reduce surgical intervention and the associated healthcare costs at the EoL, this requires randomized prospective evaluation.

Standardized, validated methods to define (1) treatment intent in the context of an individual’s cancer care and (2) standardized definitions of the EoL period could facilitate high-quality epidemiological analysis and assessment of the care received by patients with cancer at the EoL. Better systematic recording of invasive procedural intent and magnitude (major or minor), with consistent time periods from identification of incurable disease to death has the potential to have significant practice-changing ramifications.

## Supplementary Material
