# Supplementary material for: Surgical Treatment Intensity at the End of Life in Patients With Cancer: A Systematic Review
Source: Ann Surg Open. 2024 Nov 12;5(4):e514. doi: 10.1097/AS9.0000000000000514 (PMC11661707; doi:10.1097/AS9.0000000000000514)
Supplement: Supplementary file 1 [file as9-5-e514-s001.pdf]

Inclusion

Defined end of life time frame y/n

Measures invasive procedures y/n

Population includes patients with malignant neoplasm y/n

Cohort, case series, case control, ecological or audit study design y/n

Exclusions

Population <18 years old y/n

Non melanoma skin cancer y/n

Haematological malignancy y/n

RCT /review/commentary/protocol y/n

Data extraction

Article characteristics

1. First author free text
2. Year integer
3. Journal free text
4. Funding stated y/n
    a. Funding body free text if yes

study characteristics

5. Design 1 cohort 2 case control 3 ecological study 4 clinical audit
6. Scale 1 multinational 2 national 3 multicentre 4 single centre 5 other
7. Countries included (list all)
    a. Country 1 Free text
    b. Country 2 Free text
    c. Country 3 Free text
    d. Country 4 Free text
    e. Country 5 Free text
8. Total number of study participants integer
9. Number of decedents integer
10. Female sex (decedents) integer
11. Population 1 Mixed cancer and not cancer 2 Only cancer
12. Population: types of cancer 1 all cancer 2 mixed selected cancers 3 breast 4 lung 5 colorectal 6 prostate 7 other GI 8 other
13. How many types of cancer were included? Integer or unknown
14. For cohort/ecological/audit designs
    a. Inclusion criteria Free text
    b. Exclusion criteria Free text

15. For case control
    a. Definition of cases Free text
    b. Definition of controls Free text
16. Number of EoL time periods studied integer
17. EoL period 1 1 <= 2 weeks 2 <=1 month 3 <=6 months 4 <= 1 year
18. EoL period 2 1 <= 2 weeks 2 <=1 month 3 <=6 months 4 <= 1 year
19. EoL period 3 1 <= 2 weeks 2 <=1 month 3 <=6 months 4 <= 1 year
20. EoL period 4 1 <= 2 weeks 2 <=1 month 3 <=6 months 4 <= 1 year
21. EoL period 5 1 <= 2 weeks 2 <=1 month 3 <=6 months 4 <= 1 year
22. Clinical and demographic variables measured
    a. Age y/n
        i. Age categorisation (1 mean 2 median 3 age group 4 elderly 5 other)
    b. Sex y/n
    c. Ethnicity y/n
    d. Comorbidity y/n
        i. Categorisation used (1 Charlson 2 other)
        ii. If other, state text
    e. Deprivation y/n
    f. Income y/n
    g. Geographical region y/n
        i. Level of geographical measurement (1 trust 2 alliance 3 health authority 4 county 5 country 6 other or combined)
        ii. If other/combined, state text
    h. Cancer type y/n
    i. Cancer histology y/n
    j. Other 1 y/n
    k. Other 2 y/n
    l. Other 3 y/n
    m. Other 4 y/n
    n. Other 5 y/n

Methodological quality – Hoy et al

Was the study's target population a close representation of the national population in relation to relevant variables? y/n

Was the sampling frame a true or close representation of the target population? y/n

Was some form of random selection used to select the sample, OR was a census undertaken? y/n

Was the likelihood of nonresponse bias minimal? y/n

Were data collected directly from the subjects (as opposed to a proxy)? y/n

Was an acceptable case definition used in the study? y/n

Was the study instrument that measured the parameter of interest shown to have validity and reliability? y/n

Was the same mode of data collection used for all subjects? y/n

Was the length of the shortest prevalence period for the parameter of interest appropriate? y/n

Were the numerator(s) and denominator(s) for the parameter of interest appropriate? y/n

Summary item on the overall risk of study bias High/Moderate/Low

Surgical intensity

23. What invasive procedures are measured? Tick all that apply
    a. Any invasive procedure (procedures not specified) y/n
    b. Specified group of procedures (e.g. neurosurgical procedures) y/n
    c. Specific invasive procedures (e.g. ileostomy formation) y/n
    d. Other y/n

24. What is the total number of measures of surgical treatment intensity integer
    a. Measure 1 Free text
    b. Measure 2 Free text
    c. Measure 3 Free text
    d. Measure 4 Free text
    e. Measure 5 Free text
    f. Measure 6 Free text
    g. Measure 7 Free text
    h. Measure 8 Free text
    i. Measure 9 Free text
    j. Measure 10 Free text
    k. Measure 11 Free text
    l. Measure 12 Free text
    m. Measure 13 Free text
    n. Measure 14 Free text
    o. Measure 15 Free text
    p. Measure 16 Free text
    q. Measure 17 Free text
    r. Measure 18 Free text
    s. Measure 19 Free text
    t. Measure 20 Free text
    u. Measure 21 Free text
    v. Measure 22 Free text
    w. Measure 23 Free text
    x. Measure 24 Free text
    y. Measure 25 Free text
    z. Measure 26 Free text

25. How is surgical treatment intensity measured
    a. Rates of procedures y/n
    b. Frequency of procedures y/n
    c. Proportions of patients receiving procedures y/n
    d. OR/RR y/n

e. Costs y/n
f. Other y/n
i. If other, state text

26. Was surgical treatment intensity compared in any way? y/n
If yes was it
a. Compared by patient demographics (e.g age, sex etc) y/n/na
b. Compared by disease characteristics (eg. Type of cancer) y/n/na
c. Compared by type of surgery y/n/na
d. Compared by EoL time period y/n/na

27. Was geographic clinical practice variation measured? y/n

28. Narrative summary of clinical results Free text

29. Narrative summary of health economic results Free text
