# Supplementary material for: Surgical Treatment Intensity at the End of Life in Patients With Cancer: A Systematic Review
Source: Ann Surg Open. 2024 Nov 12;5(4):e514. doi: 10.1097/AS9.0000000000000514 (PMC11661707; doi:10.1097/AS9.0000000000000514)
Supplement: Supplementary file 2 [file as9-5-e514-s002.pdf]

<u>Supplementary Table 1</u> – Funding for Studies

Author	Year of Publication	Funding
**Axelsson**	**1997**	Not stated
**Barnato**	**2004**	National Institute on Aging, Homer Laughlin Endowment, Agency for Healthcare Research and Quality to Stanford University
**Braga**	**2007**	Not stated
**Kwok**	**2011**	None
**Barnet**	**2013**	Support was provided solely from institutional and/or departmental sources.
**Lopez**	**2013**	Not stated
**Kwok**	**2014**	National Institutes of Health grants
**Alturki**	**2014**	Boursier Award – Fonds de recherché Quebec en sante
**Obermeyer**	**2014**	National Institutes of Health grant, National Cancer Institute grant and Healthcare Research and Quality grant
**Collins**	**2014**	Translational cancer research Grant [EO109_29] from the Victorian Cancer Agency of the Victorian State Government Department of Health
**Krell**	**2015**	National Institutes of Health, the Agency for Healthcare Research and Quality, and the American Cancer Society
**Shiovitz**	**2015**	Hutchinson Institute for Cancer Outcomes Research
**Wu**	**2015**	Astex (Inst), Kevelt (Inst), Eisai (Inst), Array (Inst)
**Barnato**	**2015**	National Institute of Aging Grant
**Du**	**2015**	Agency for Healthcare Research and Quality (R01-HS018956) grant and in part by a grant from the Cancer Prevention and Research Institute of Texas (RP130051).
**Liu**	**2016**	Bureau of Health Promotion, Taiwan, with partial support from National Health Research Institute
**Ong**	**2016**	
**Daly**	**2016**	The production of this manuscript was funded by the Conquer Cancer Foundation Mission Endowment. Supported by a T-32 National Institutes of Health /National Cancer Institute basic research training grant in medical oncology (B.D.)
**Triplett**	**2017**	XBiotech (Inst), AstraZeneca (Inst), Merck (Inst)
**Tukey**	**2017**	Merit Review Award from the Health Services Research and Development Service, United States Department of Veterans Affairs.
**Schwartz**	**2018**	Medivation LLC, a Pfizer, Inc. and Astellas Pharma Inc
**Jang**	**2018**	None
**Sompratthana**	**2018**	This study was funded by the Ratchadapisek Sompoch Fund, Faculty of Medicine, Chulalongkorn University RA60/059.
**Urban**	**2018**	
**Kuo**	**2019**	None
**De Man**	**2019**	Funded by the National Health Care Institute of the Netherlands.
**Niteki**	**2019**	The authors have not declared a specific grant for this research from any funding agency in the public, commercial or not-for- Profit sectors.
**Wächter**	**2020**	None

**Fond**	**2020**	This work was funded by AP-HM (Assistance Publique des Hôpitaux de Marseille) and Aix-Marseille University.
**Fond**	**2020**	This work was funded by Assistance Publique des Hôpitaux de Marseille and Aix-Marseille University. The authors declare no conflict of interest.
**Martins-Brance**	**2020**	This work was supported by the Calouste Gulbenkian Foundation as part of the DINAMO Project (grant number 127 988 to BG); and the Núcleo Regional do Sul – Liga Portuguesa Contra o Cancro (43/2015 and 35/2016 to DM-B).
**Viprey**	**2020**	No specific grant from any funding agency, commercial or not-for-profit sectors
**Fond**	**2021**	This research received no specific grant from any funding agency, commercial or non-profit sectors
**Schmitz**	**2021**	Data collection of the SONABRE Registry is funded by the Netherlands Organization for Health Research and Development; Novartis BV; Roche; Pfizer, Eli Lilly
**Ullgren**	**2021**	This was supported by grants from the Cancer Research Foundation in Northern Sweden
**Wang**	**2022**	This research received no external funding
**Chiaruttini**	**2022**	This study was supported by contributions from the Ministry of Health of the Lombardy Region as part of the EPIFARM-Pharmaco-Epidemiology Agreement between the Istituto di Ricerche Farmacologiche Mario Negri IRCCS and the Lombardy Region
**Broekman**	**2022**	This research did not receive any specific grant from funding agencies in the public, commercial, or not-for-profit sector
**Vestergaard**	**2023**	Independent Research Fund Denmark [grant number 4004-00609B], Fonden af 1870 [grant number 200652], C.C. Klestrup & Hustru Henriette Klestrups Mindelegat [grant number 10761], Fabrikant Einar Willumsens Mindelegat [grant number 131 217] and Helga og Peter Kornings Fond [grant number 472123-002-40].
