# Supplementary material for: Surgical Treatment Intensity at the End of Life in Patients With Cancer: A Systematic Review
Source: Ann Surg Open. 2024 Nov 12;5(4):e514. doi: 10.1097/AS9.0000000000000514 (PMC11661707; doi:10.1097/AS9.0000000000000514)
Supplement: Supplementary file 3 [file as9-5-e514-s003.pdf]

Supplementary Table 2 – Invasive procedure by clinical endpoint

| Study | Cancer Type | Time from diagnosis | Time from metastatic cancer diagnosis | Time from palliative care/hospice referral | Time since last admission |
|---|---|---|---|---|---|
| **Kwok 2014** | **Mixed** | 96.20% | - | - | - |
| **Krell 2015** | **Colorectal** | - | 15,847-48,525 (18.8%-57.7%) | - | - |
| **Obermeyer 2014** | **Mixed** | - | - | 7,057' (38.9%) | - |
| **Shiovitz 2015** | **Mixed** | - | 46,727 (61.3%) | - | - |
| **Schwartz 2018** | **Breast** | 743 (89.7%) | 152 (36.5%) | - | - |
| **Triplett 2017** | **Mixed** | - | - | 44-342 (0.7%-5.2%) | - |
| **Barnato 2015** | **Mixed** | - | - | - | 1,326-15,632 (1.6%-18.8%) |
| **Wächter, 2020** | **Thyroid** | - | - | 36-42 (85.7%-100%) | - |
| **Collins, 2014** | **Neurological** | 108-509 (16-75%) | - | - | 61-68 (28-31%)* |
| **Sompratthana, 2018** | **Gynaecological** | | | | 8.80% |
