# Supplementary material for: Surgical Treatment Intensity at the End of Life in Patients With Cancer: A Systematic Review
Source: Ann Surg Open. 2024 Nov 12;5(4):e514. doi: 10.1097/AS9.0000000000000514 (PMC11661707; doi:10.1097/AS9.0000000000000514)
Supplement: Supplementary file 4 [file as9-5-e514-s004.pdf]

Supplementary Table 3 – Risk of Bias Assessment

		Was the study's target population a close representation of the national population in relation to relevant variables?	Was the sampling frame a true or close representation of the target population?	Was some form of random selection used to select the sample, OR was a census undertaken?	Was the likelihood of nonresponse bias minimal?	Were data collected directly from the subjects (as opposed to a proxy)?	Was an acceptable case definition used in the study?	Was the study instrument that measured the parameter of interest shown to have validity and reliability?	Was the same mode of data collection used for all subjects?	Was the length of the shortest prevalence period for the parameter of interest appropriate?	Were the numerator and denominator for the parameter of interest appropriate?	Risk of bias
**Axelsson**	**1997**	N	Y	N	Y	N	Y	Y	Y	Y	Y	Moderate
**Barnato**	**2004**	N	Y	Y	Y	N	Y	Y	Y	Y	Y	Low
**Braga**	**2007**	N	Y	Y	Y	N	Y	Y	Y	Y	Y	Low
**Kwok**	**2011**	Y	Y	Y	Y	N	Y	Y	Y	Y	Y	Low
**Barnet**	**2013**	N	Y	Y	Y	N	Y	Y	Y	Y	Y	Moderate
**Lopez**	2013	N	N	N	Y	N	Y	N	Y	Y	Y	Moderate
**Kwok**	**2014**	N	Y	Y	Y	N	Y	Y	Y	Y	Y	Low
**Alturki**	**2014**	N	Y	Y	Y	N	Y	Y	Y	Y	Y	Low
**Collins**	**2014**	N	Y	Y	Y	N	Y	Y	Y	Y	N	Moderate
**Obermeyer**	**2014**	N	Y	Y	Y	N	Y	Y	Y	Y	Y	Moderate
**Du**	**2015**	N	Y	Y	Y	N	Y	Y	Y	Y	Y	Low
**Krell**	**2015**	N	Y	Y	Y	N	Y	Y	Y	Y	Y	Moderate
**Wu**	**2015**	N	N	Y	Y	Y	Y	Y	Y	Y	N	High
**Shiovitz**	**2015**	N	Y	Y	Y	N	Y	Y	Y	Y	N	High
**Barnato**	**2015**	N	Y	Y	Y	N	Y	Y	Y	Y	Y	Low
**Ong**	**2016**	N	N	Y	Y	N	Y	N	Y	Y	Y	High
**Daly**	**2016**	N	N	Y	Y	N	N	N	Y	Y	N	Moderate
**Liu**	**2016**	N	Y	Y	Y	N	Y	Y	Y	Y	Y	Low
**Tukey**	**2017**	N	Y	Y	Y	N	Y	Y	Y	Y	Y	Moderate
**Triplett**	**2017**	N	Y	Y	Y	N	Y	Y	Y	Y	Y	Low
**Schwartz**	**2018**	N	Y	Y	Y	N	Y	y	y	Y	Y	Low
**Sompratthana**	2018	N	N	N	Y	N	Y	N	Y	Y	N	Moderate

**Urban**	2018	Y	Y	N	Y	N	Y	Y	Y	Y	Y	Low
**Jang**	**2018**	N	N	N	Y	Y	Y	Y	Y	Y	Y	Moderate
**Niteki**	2019	N	N	N	Y	N	Y	N	Y	Y	Y	Moderate
**Kuo**	**2019**	N	Y	Y	Y	N	Y	Y	Y	Y	Y	Low
**De Man**	**2019**	N	Y	Y	Y	N	Y	Y	Y	Y	Y	Low
**Wächter**	**2020**	N	Y	Y	Y	N	Y	Y	Y	Y	Y	Moderate
**Fond (JAD)**	**2020**	N	Y	Y	Y	N	Y	Y	Y	Y	Y	Low
**Fond (APS)**	**2020**	N	Y	Y	Y	N	Y	Y	Y	Y	Y	Moderate
**Martins-Brance**	**2020**	N	Y	Y	Y	N	Y	N	Y	Y	Y	Moderate
**Viprey**	**2020**	N	Y	N	Y	N	Y	N	Y	Y	Y	Moderate
**Fond**	**2021**	N	Y	Y	Y	N	Y	Y	Y	Y	Y	High
**Schmitz**	**2021**	N	N	N	Y	N	Y	N	Y	Y	Y	Moderate
**Ullgren**	**2021**	Y	Y	N	Y	N	Y	N	Y	Y	Y	Moderate
**Wang**	**2022**	N	Y	N	N	N	Y	Y	Y	Y	Y	Low
**Chiaruttini**	**2022**	Y	Y	N	Y	N	Y	N	Y	Y	Y	Low
**Broekman**	**2022**	Y	N	N	Y	N	Y	Y	Y	Y	Y	Moderate
**Vestergaard**	**2023**	N	Y	Y	N	Y	N	Y	Y	Y	Y	Moderate
